# The venom of *Cyriopagopus schmidti* spider contains a natural huwentoxin-IV analogue with unexpected improved analgesic potential

**DOI:** 10.3389/fphar.2025.1566312

**Published:** 2025-04-10

**Authors:** Aurélie Antunes, Jérôme Montnach, Kuldip Khakh, Ludivine Lopez, Baptiste Thomas, Barbara Ribeiro Oliveira-Mendes, Lucie Jaquillard, Denis Servent, Rémy Béroud, Charles J. Cohen, Evelyne Benoit, Michel De Waard

**Affiliations:** ^1^ Université Paris-Saclay, CEA, Département Médicaments et Technologies pour la Santé (DMTS), Service d’Ingénierie Moléculaire pour la Santé (SIMoS), EMR Centre National de la Recherche Scientifique, Gif-sur-Yvette, France; ^2^ Smartox Biotechnology, Saint-Egrève, France; ^3^ Nantes Université, Centre National de la Recherche Scientifique, Institut National de la Santé et de la Recherche Médicale, l’institut du thorax, Nantes, France; ^4^ Department of Biology, Xenon Pharmaceuticals, Burnaby, BC, Canada; ^5^ Smartbioscience-Peptide, Saint-Egrève, France; ^6^ LabEx “Ion Channels, Science and Therapeutics”, Valbonne, France

**Keywords:** spider venom peptide, huwentoxin-IV, Na_v_1.7, pain treatment, dorsal root ganglion neuron, SAR study, automated high-thoughput patch-clamp

## Abstract

The venom of *Cyriopagopus schmidti* spider has been extensively investigated, thereby allowing the identification of numerous new natural peptides. Many of these peptides are active on ion channels and several of them occur from post-translational processing. In order to further identify new entities, we screened this venom against five different human voltage-gated sodium (hNa_v_) channels. We illustrate the unusual richness of this venom in targeting this wide variety of hNa_v_ channels. We confirm the identity of previously discovered peptides active on these ion channels type (huwentoxin (HwTx)-I, HwTx-II and HwTx-IV), indicating the efficacy of the screening process by automated patch-clamp. We also identified a novel analogue of HwTx-IV that differs by the absence of amidation and the presence of an extra C-terminal Gly residue. Interestingly, this analogue is less potent than HwTx-IV itself in blocking hNa_v_1.7 in cell lines, but turns out to be significantly more potent in TTX-sensitive dorsal root ganglia neurons. Because of this unexpected finding, this novel analogue turns out to be a more potent analgesic than HwTx-IV itself without presenting most of the Na_v_1.6-related toxic effects of HwTx-IV.

## 1 Introduction

Spider venoms have been proficient in providing toxins active on voltage-gated sodium (Na_v_) channels (Escoubas, 2006). Identified peptides are 28–79 amino acid residues in length with most of them displaying a typical inhibitor cystine fold that is frequently hard to reproduce during chemical synthesis. Up to now, 1,576 natural peptides have been identified and categorized into 12 subfamilies from NaSpTx-1 to NaSpTx-12 (Klint et al., 2012) compiled into a specific database (https://arachnoserver.qfab.org/mainMenu.html) (Pineda et al., 2018). The same database provides their amino acid sequences and recapitulates known ion channel activities. Amino acid sequences, secondary structures and disulfide bridging patterns all contribute to the functional diversity of these peptides. Post-translational modifications (PTM) occurring in the venom gland of venomous species represents another leading cause for pharmacological diversity. In this respect, the venom glands of marine cone snails are leading examples of PTM of natural peptides (Buczek et al., 2005a). Cone snail peptide PTM include classical amidation of the C-terminus, pyroglutamation of the N-terminus (often witnessed by the impossibility to perform Edman degradation), *O*-glycosylation of Thr or Ser residues, phosphorylation, γ-carboxylation of Glu residue, epimerization to a D-amino acid (Buczek et al., 2005b), hydroxylation of a Pro residue, cyclisation of Gln, bromination of Trp, and sulfation of a Tyr residue. Glycosylation of contulakin-G has been associated to a 10-fold increase in activity of the peptide (Craig et al., 1999). However, the functional impact of all these PTM is only emerging. Interestingly, they are not limited to cone peptides only. For instance, phosphorylation of maurocalcin, a scorpion venom peptide, by protein kinase A converts the peptide from an allosteric activator to a ryanodine receptor blocker (Ronjat et al., 2016). To extend these observations, we dedicated our attention to peptides from spider venoms with a particular focus on *Cyriopagopus schmidti* (previously known as *Haplopelma schmidti* or *Selenocosmia huwena*) tarentula venom for which several peptides of interest have been discovered (Liang, 2004). Huwentoxin-IV (HwTx-IV) has been the most investigated one because of its high affinity for human (h)Na_v_1.7, an interesting target for pain treatment (Peng et al., 2002; Xiao et al., 2008; Xiao et al., 2010; Xiao et al., 2011; Minassian et al., 2013; Revell et al., 2013; Sermadiras et al., 2013; Liu et al., 2014; Agwa et al., 2017; Rahnama et al., 2017; Agwa et al., 2020; Neff et al., 2020; Lopez et al., 2021). It is also termed μ-theraphotoxin-Hs2a in the ArachnoServer database (Pineda et al., 2018). It was initially discovered by purification and sequencing of a set of seven peptides (HwTx-I, HwTx-II, HwTx-IIIa, HwTx-IV, HwTx-V, HwTx-VII and SHL-I; the last being a lectin rather than a toxin) from this spider venom gland (Liang et al., 1993; Peng et al., 2002), and its sequence confirmed by cloning efforts (Diao et al., 2003). All cDNA sequences encode for a signal peptide of 21–24 amino acids and a propeptide region of 27–29 amino acids in length. Both sequences are cleaved, firstly with a signal peptidase, thanks to a consensus cleavage point, and, secondly, by a propeptide processing enzyme recognizing an EER sequence to produce the mature peptides (Diao et al., 2003). Comparison of the protein sequences predicted to occur from the cDNA, until termination of translation with a stop codon, and the corresponding identified peptides in the venoms, highlights that several interesting post-translational processing should occur. HwTx-IIIa and SHL-I were produced with an additional Arg-Arg dipeptide, while HwTx-V comprised an additional Lys residue. All these amino acids were thus removed by post-translational processing. Nevertheless, two other analogues were detected: SHL-Ia and HwTx-IIIb that differ from their SHL-I and HwTx-III counterparts by the loss of a Trp residue at the C-terminus. Additional sequence divergence among analogous toxins may arise from ambiguous recognition of the cleavage site by the precursor processing enzyme, thereby generating for instance HwTx-VIII that has lost a Leu residue at the N-terminus of HwTx-II. Concerning HwTx-IV, the coding sequence predicts an additional Gly-Lys dipeptide at the C-terminus that is thus also processed (Diao et al., 2003). In addition, analyses by mass spectrometry (MS) indicated that HwTx-IV is amidated at the C-terminus (Diao et al., 2003), a PTM that should occur after removal of the Gly-Lys dipeptide. Finally, a second form of HwTx-IV has been detected in the venom of *C. schmidti* where the Glu residue is modified into a pyroglutamic acid residue (pHwTx-IV) (Rong et al., 2013). A pyroglutamate is thought to stabilize against degradation by aminopeptidases. Alternatively, it may stabilize the toxin by neutralizing the N-terminal basicity through the action of glutaminyl cyclisation (Schilling et al., 2008). At the functional level, however, pHwTx-IV was shown to better trap the voltage sensor of Na_v_1.7, presumably by enhancing its affinity for this target.

Among the toxins discovered within the venom of *C. schmidti* several of them act on ion channels, while others have undefined targets or simply are dubbed with insecticidal activities. HwTx-I acts on N-type calcium channels (Peng et al., 2001), HwTx-IV inhibits tetrodotoxin (TTX)-sensitive (TTX-S) sodium channels (Peng et al., 2002), and pHwTx-IV preserves the selectivity profile of HwTx-IV but differs from it by a diminished depolarization-induced dissociation of the toxin from the channel (Rong et al., 2013). HwTx-II, HwTx-IIIa, HwTx-VII, and HwTx-V all appear to have insecticidal activities (Zhang et al., 2003). SHL-I has lectin activity and favors hemagglutination (Liang and Pan, 1995). All these earlier reports indicate that the *C. schmidti* venom is rich in bioactive toxins modulating ion channels and that post-translational processing participates actively in the generation of interesting peptide variants but of unknown activity. To get a grip on how many diverse toxins are generated in the venom of this spider species, we decided to screen for active compounds onto a set of five different hNa_v_ channels and identified a few ones that may highlight the role of these post-translational processing events on toxin pharmacology. By doing so, we identified a new naturally-occurring analogue of HwTx-IV (HwTx-IV G_COOH_) that displays an interesting increase in activity on mouse Na_v_1.7 (mNa_v_1.7) of dorsal root ganglia (DRG) neurons, compared to HwTx-IV, while concomitantly producing analgesic effects at lower threshold without apparent toxic effects at the higher doses. Interestingly, the peptide was less active than HwTx-IV on hNa_v_1.7 channels expressed in cell lines highlighting that structure-function studies performed on human embryonic kidney (HEK)-293 or Chinese hamster ovary (CHO) cell lines expressing Na_v_ channels outside their native environment can fail to select for the most potent analgesic peptides.

## 2 Materials and methods

### 2.1 Venom fractionation and toxin purification

21 mg of the venom from the *C. schmidti* spider (also known as “Chinese bird spider” or “Chinese earth tiger”, *Ornithoctoninae* family) were separated within 64 fractions using a Phenomenex semi-preparative RP-HPLC C18 column (Proteo Jupiter, 4 µm of 10 mm ID x 250 mm L) attached to an LC-20AD HPLC (Shimadzu). One-10th of these fractions were used for screening purposes, while the remaining 90% were kept for further purification of active compounds if needed. Positive fractions were further sub-fractionated using cation exchange chromatography with a TOSOH Bioscience column (TSK gel SP-STAT, 7 μm, 4.6 mm ID x 10 cm L, TOSOH Bioscience, Germany) onto an Agilent 1260 HPLC (Agilent technologies). All purified peptides resulting from this sub-fractionation were tested at concentrations similar to the one present in the primary fractions.

### 2.2 Venom screening using a Na^+^ fluorescent indicator

The fluorescence 384-well HTS assay used on venom fractions were run in antagonist and agonist modes on stable cell lines expressing either hNa_v_1.1, hNa_v_1.2, hNa_v_1.5, hNa_v_1.6 or hNa_v_1.7, coexpressed with Kir1.1 to enable changes in membrane potential by altering extracellular potassium concentrations. Veratridine was used to promote sodium influx and test for antagonists, while by omitting veratridine, the assay tests for the presence of channel activators. The protocol for testing venom fractions for Na_v_ channel inhibition was setup as follows: *(i)* cells were loaded with a Na^+^-sensitive dye (Asante Natrium Green-2, ANG2) in the absence of Na^+^, *(ii)* cells were pre-incubated with primary venom fractions diluted 100-fold in the presence of veratridine for 1 h, *(iii)* cells were challenged with a buffer containing Na^+^ and a high K^+^ concentration, and *(iv)* the Na^+^ influx was measured. The same protocol was used with purified compounds at concentrations that matched the concentrations used for primary screening. More details on cell cultures and protocol have been reported elsewhere (Lopez et al., 2023).

### 2.3 Primary structure determination of venom peptides

The amino acid sequences of the hit peptides were obtained by a combination of Edman degradation and *de novo* MS/MS sequencing. Purified peptides were resuspended in 100 mM ammonium bicarbonate (pH 8.0), reduced during 1 h with 17 mM TCEP at 55°C and alkylated during 1 h with 24 mM iodoacetamide (room temperature (RT) in the dark). For Edman degradation, amino acid sequence determination was performed using an Applied Biosystems gas-phase sequencer model 492. For liquid chromatography-electrospray ionization-tandem mass spectrometry (LC-ESI-MS/MS) data, the reduced/alkylated peptides were digested by using trypsin or V8 proteases (1:10 ratio (enzyme/peptide, w/w), overnight incubation at 37°C). A Waters Q-TOF Xevo G2S mass spectrometer equipped with an Acquity UHPLC system and Lockspray source was used for the acquisition of the LC-ESI-MS and LC-ESI-MS/MS data.

### 2.4 Chemical syntheses of *Cyriopagopus schmidti *spider peptides

All peptides were assembled stepwise using 2-chlorotrityl chloride resins by solid-phase fmoc chemistry on a Symphony Synthesizer (Protein technologies Inc.). Resin cleavage of the peptides and deprotection were conducted with 92.5% (vol) TFA, 2.5% H_2_O and scavengers (1,3-dimethoxybenzene (2.5%) and triisopropylsilane (2.5%)). Next, the peptides were purified to homogeneity by C18 RP-HPLC on a Jupiter Proteo column (Phenomenex, 4 μm, 21.2 mm ID x 250 mm L) using an Agilent Technologies preparative HPLC (1,260 Infinity). All peptides were folded/oxidized in 50 mM Tris-HCl, pH 8.3 during 72 h, and purified to homogeneity (>99% purity) using RP-HPLC, again with the Jupiter Proteo column. The molecular masses of the peptides were determined by LC-ESI quadrupole time-of-flight (QTOF) MS.

### 2.5 Toxins

Lyophilized synthetic (s) HwTx-IV G_COOH_ (molecular weight of 4164.82 Da, purity rate >97%, Smartox Biotechnology, Saint-Egrève, France), HwTx-IV (molecular weight of 4107.20 Da, purity rate >97%; Smartox Biotechnology, Saint-Egrève, France) and TTX citrate (molecular weight of 319.27 Da, purity rate >98%; Sigma-Aldrich, Saint-Quentin Fallavier, France) were dissolved in PBS +0.1% BSA (for sHwTx-IV G_COOH_ and HwTx-IV) or with 10% phosphate buffer (for TTX), to give stock solutions of 1 mM, 6.1 mM and 6 mM, respectively, and stored at −20°C. Successive dilutions were then performed in the adequate medium (PBS +0.1% BSA or standard physiological medium), prior to experiments, to give the final toxin concentrations/doses indicated in the text.

### 2.6 Cell cultures for automated patch-clamp recordings

CHO cells stably expressing hNa_v_1.7 were cultured in Dulbecco’s Modified Eagle’s Medium (DMEM) supplemented with 10% foetal calf serum, 1 mM pyruvic acid, 4 mM glutamine, 10 U/mL penicillin and 10 μg/mL streptomycin (Gibco, Grand Island, NY), and incubated at 37°C in a 5% CO_2_ atmosphere. For electrophysiological recordings, cells were detached with trypsin and floating single cells were diluted (∼300,000 cells/mL) in medium containing (in mM): 4 KCl, 140 NaCl, five glucose, 10 2-[4-(2-hydroxyethyl)piperazin-1-yl]ethanesulfonic acid (HEPES, pH 7.4, osmolarity 290 mOsm). Similar culturing conditions were used for HEK-293 cell lines expressing hNa_v_1.1, hNa_v_1.2, hNa_v_1.3, hNa_v_1.5 and hNa_v_1.6 channels. The plasmid pcDNA3.1+/C-(K)-DYK (Genscript, Piscataway, New Jersey, USA) was used for transient expression of mNa_v_1.7 in HEK-293T cells using the Maxcyte electroporation system.

### 2.7 Automated patch-clamp experiments

Whole-cell recordings using the automated patch-clamp system SyncroPatch 384 PE (Nanion Technologies, Munich, Germany) were used to investigate the effects of HwTx-IV and sHwTx-IV G_COOH_ on HEK-293T or CHO cells stably expressing hNa_v_1.1, hNa_v_1.2, hNa_v_1.6 or hNa_v_1.7, or on HEK293 cells electroporated with mNa_v_1.7. Chips with single-hole and high-resistance 5.14 ± 0.02 MΩ (*n* = 384) were used for HEK-293 cell recordings. Only cells with seal resistance above 500 MΩ and Rs values <15 MΩ were kept for analyses. All Rs values were compensated at 90%. Voltage pulses and whole-cell recordings were achieved using the PatchControl384 v1.5.3 software (Nanion Technologies, Munich, Germany) and the Biomek v1.0 interface (Beckman Coulter, Villepinte, France). Prior to recordings, dissociated cells were shaken at 60 rpm in a cell hotel reservoir at 10°C. After cell catching, sealing, whole-cell formation, liquid application, recording, and data acquisition were all performed sequentially and automatically. The intracellular solution contained (in mM): 10 KCl, 110 kF, 10 NaCl, 1 MgCl_2_, 1 CaCl_2_, 10 Ethylene Glycol Tetraacetic Acid (EGTA) and 10 HEPES (pH 7.2, osmolarity 280 mOsm), and the extracellular solution (in mM): 140 NaCl, 4 KCl, 2 CaCl_2_, 1 MgCl_2_, five glucose, and 10 HEPES (pH 7.4, osmolarity 298 mOsm). Whole-cell experiments were done at −100 mV holding potential, while currents triggered at 0 mV test potential were sampled at 10 kHz (sweeps every 8 s). Compounds were prepared at various concentrations in the extracellular solution supplemented with 0.3% BSA and distributed in 384-well plates according to a pre-designed template. Compound solutions were diluted three times in the patch-clamp recording well by adding 30–60 μL external solution to reach the final reported concentration and the test volume of 90 μL. The percentage of current inhibition was measured after a 13-min application time. A single concentration of peptide was tested on each cell for building the full-inhibition curves.

### 2.8 Primary cultures of DRG neurons

Adult female Swiss mice (*Mus musculus*, 8–14 weeks of age and 25–45 g body weight) were purchased from Janvier Elevage (Le Genest-Saint-Isle, France) and acclimatized for at least 48 h before experiments at the CEA animal facility. They were housed in a 12-h light/dark cycle and controlled temperature room, six-wise in cages containing bedding and a cardboard tube for environmental enrichment and were allowed free access to water and food. The experiments involving animals were conducted in compliance with the guidelines established by the French Council on animal care “Guide for the Care and Use of Laboratory Animals” (Decree 2013-118), and the experimental protocols were approved on 20 July 2020 by the French General Directorate for Research and Innovation (project APAFIS#26651-2020072011192542v1 authorized to E.B.). After mouse anesthesia (by 2.0%–2.5% isoflurane inhalation) and euthanasia (by cervical vertebrae dislocation), the DRG were dissected from intervertebral foramina of the vertebrate column, placed in iced-Ham’s F-12 medium (Sigma-Aldrich) and enzymatically dissociated with collagenase type IA (2 mg/mL; Sigma-Aldrich) and dispase (5 mg/mL; Gibco, Thermo Fisher Scientific, Villebon-sur-Yvette, France). The neurons were cultured under standard conditions (37°C, 95% air and 5% CO_2_) on 12-mm glass coverslips placed in a 24-wells plate coated with 100 μg/mL of murin laminin and 10 μg/mL of poly-D-lysine (Sigma-Aldrich). The culture medium was composed of a Neurobasal A medium (Gibco) added with Dulbecco’s PBS w/o CaCl_2_ and MgCl_2_ (1.68%; Gibco), BSA (16.83 μg/mL; Sigma-Aldrich), corticosteron (214.85 nM; Sigma-Aldrich), T3 hormone (56.06 nM; Sigma-Aldrich), horse serum (5%; Gibco), penicillin/streptomycin (47.64 U/mL; Gibco), nerve growth factor (83.33 ng/mL; Sigma-Aldrich), N2 supplement (3.18x; Gibco) and L-glutamine (1.90 mM; Sigma-Aldrich). One day later, cytosine β-D-arabinofuranoside (2 μM; Sigma-Aldrich) was added to the medium to inhibit astrocyte proliferation. Experiments were carried out within 4–8 days after neuron dissociation.

### 2.9 Manual patch-clamp recordings of DRG neurons

Prior to recording, DRG neurons, plated on coverslips, were transferred for a minimum of 30 min at 37°C in 35-mm Petri dishes filled with a standard physiological medium of the following composition (in mM): 134 NaCl, 3 KCl, 1 CaCl_2_, 1 MgCl_2_, 10 D-glucose, 10 tetraethylammonium chloride (TEA), 0.1 CdCl_2_, 0.1% BSA and 10 HEPES (pH 7.35, adjusted with NaOH; 302 mosm), and then in the recording bath filled with the standard physiological medium. Whole-cell manual patch-clamp experiments were performed by using a MultiClamp 700B integrating patch-clamp amplifier and the pClamp v10.6 software (Molecular Devices, Sunnyvale, CA, USA). The signals, acquired at a 4-kHz sample rate, were filtered at 2 kHz with a low-pass Bessel filter and digitized with the aid of a computer equipped with an analog-to-digital converter (Digidata-1440A model; Molecular Devices). The patch-clamp pipettes were filled with a medium composed of (in mM) 90 CsCl, 40 CsMeSO_3_, 10 NaCl, 2 MgCl_2_, 2 EGTA, 4 Na_2_ATP and 10 HEPES (pH 7.32, adjusted with CsOH; 292 mosm), and had a resistance of 3.3 ± 0.1 MΩ (n = 99) in the standard physiological medium. Mean access resistance was 8.3 ± 0.3 MΩ (n = 91 cells) and was compensated at 80%. A fast solution application system allowed changing the solution (standard physiological medium supplemented or not with a given toxin concentration) around the recorded cell within a few seconds. The toxin effects were evaluated by perfusing neurons with the standard physiological medium containing various concentrations of a given toxin or by directly adding a toxin solution of a given concentration (maximum volume of 20 μL) to the standard physiological solution bathing the neurons (1 mL volume). To obtain information on the reversibility of toxin effects (if they were significant), the neurons were perfused with a standard physiological solution devoid of toxin. The experiments were carried out at constant room temperature (20°C). The neurons were maintained at a holding potential of −80 mV, and currents were elicited at a frequency of 0.5 Hz by 50-m test-pulses to −20 mV preceded by 1 s pulses to −100 mV. The amplitude of the leakage current (not compensated) was −445.1 ± 48.6 pA (n = 99 neurons) and represented 7.3% of that of peak Na^+^ current (*i.e.*, −6.1 ± 0.3 nA, n = 99 neurons). Current-voltage relationships were obtained by varying test-pulses from −80 to +60 mV in 5-mV increments, and steady-state inactivation-voltage relationships by changing prepulses from −120 to −20 mV in 5 mV increments. The cell diameter (d) was determined from the cell membrane capacity (Cm in Farads) by assimilating the cell to a sphere whose area (Sm in m^2^) is Πd^2^ and assuming that the cell membrane area is directly proportional to the cell membrane capacity (Sm = Cm/c, where c represents the specific membrane capacity that does not markedly vary from 1 cell type to another and is on average 10^−2^ F/m^2^). The concentration-response relationships were established by plotting the peak current amplitude, measured in the presence of a given toxin (It) and expressed as percentage of the value obtained before toxin application (Ic), against the toxin concentration ([toxin]). The theoretical concentration-response curves were calculated from typical sigmoid nonlinear regressions through data points (correlation coefficient = *r* ^2^), according to the Hill equation (GraphPad Prism v5 software): It/Ic = 1/[1 + ([toxin]/IC_50_) n_H_], where IC_50_ is the toxin concentration necessary to inhibit 50% of the response and n_H_ the Hill number. Current kinetics were evaluated by calculating the two following activation and inactivation parameters: the time (Time_A_) of current increase from 10% to 90% (activation) and the time (Time_I_) of current decrease from 90% to 10% (inactivation). The conductance (g) was calculated according to the following equation: g = I/(V_T_–V_Na_), where I is the peak current amplitude, V_T_ the test-pulse voltage and V_Na_ the equilibrium potential of Na^+^ ions. Conductance-voltage relationships were established by plotting the conductance, expressed as percentage of the maximal conductance (gmax) calculated at large positive test-pulses, as a function of test-pulse voltage. The theoretical curves correspond to data point fits, according to the Boltzmann equation (GraphPad Prism v5 software): g/gmax = 1 – [1/(1 + exp ((V_T_–V_T50%_)/k_G_))], where V_T50%_ is the test-pulse voltage corresponding to 50% maximal conductance, and k_G_ is the slope of the curve. Steady-state inactivation-voltage relationships were established by plotting the peak current amplitude, expressed as percentage of the maximal amplitude (Imax) recorded in response to large negative pre-pulses, as a function of pre-pulse voltage (V_P_). The theoretical curves correspond to data point fits, according to the Boltzmann equation: I/Imax = 1/[1 + exp ((V_P_–V_P50%_)/k_H_)], where V_P50%_ is the pre-pulse voltage corresponding to 50% maximal peak amplitude of current and k_H_ is the slope of the curve.

### 2.10 Pain sensitivity assays in mice *in vivo*

Animal experiments were performed using adult female Swiss mice (average weight 32.3 ± 3.8 g, n = 78), as described above, and are reported in line with the ARRIVE (Animal Research: Reporting of *In Vivo* Experiments) guidelines developed in consultation with the scientific community as part of an NC3Rs initiative to improve standards of reporting the results of animal experiments, maximizing information published and minimizing unnecessary studies (Kilkenny et al., 2010; Schulz et al., 2010). The study was experimentally designed to have group sizes of at least six animals per group, with intravenous injection as an exclusion criterion. In addition, randomization and blinding (one of the two experimenters being blind to treatment group) were undertaken in all animal experiments. The sHwTx-IV G_COOH_ and HwTx-IV effects on pain sensitivity were evaluated using the automated von Frey test (tactile sensitivity) and the hot-plate test (heat sensitivity). Prior to testing, each mouse underwent a 20-min acclimation to the experimental laboratory environment (in its home cage). Then, mice were either not injected or received an intraplantar injection of PBS +0.1% BSA (vehicle) or toxin solution (maximal volume of 15 µL for the largest animal on the basis of 10 μL for a 30 g mouse) in the right hind limb, under low anaesthesia achieved by means of isoflurane inhalation (AErrane^®^, Baxter S.A.; 2.0%–2.5% during about 2 min). It should be noted that information on the peptide toxicity was obtained during these experiments through the behaviour of mice and/or death of animals and quantified by an Actimeter for sHwTx-IV G_COOH_.

### 2.11 Mechanical stimulus-induced pain

Tactile sensitivity was assessed using an automated plantar von Frey apparatus (Dynamic Plantar Aesthesiometer 37,450, Ugo Basile, Comerio, Italy). Prior to testing, each mouse was placed on a mesh grid, surrounded by a clear Plexiglas barrier with a top cover, and left to calm down for at least 15 min without probing. After the settling phase, the mouse was motionless allowing for its right hind limb to be touched by a flexible plastic fibre of a fixed diameter. The fibre was pressed through the mesh grid against the plantar surface at a right angle, and the force of application increased slowly (at the determined rate of 1.67 g.sec^-1^). The force intensity (in g) at which the animal removed its hind limb was recorded with a timer integrated into the set-up as the mean of at least five tests. A cut-off automatically occurred if the animal did not remove its hind limb when the point at which the greatest pre-set force was met, to prevent tissue damage. The force intensity was determined before and from 15 to 30 min after intraplantar injection of PBS +0.1% BSA or toxin solution.

### 2.12 Heat stimulus-induced pain

Each mouse, injected or not with PBS +0.1% BSA or toxin solution in the right hind limb, was put on a hot-plate (HCP No. 35150, Ugo Basile, Italy) set at the temperature of 55.0°C ± 0.2°C 15–30 min after limb injection. The measured parameter as the first pain-related manifestation was the latency (in seconds) for the animal to shake the right hind limb (Deuis et al., 2017). This latency, considered as a painful response to heat, was recorded simultaneously by two observers (one being blind to treatment group) with a timer integrated into the set-up. A maximal cut-off time of 30 s was used to prevent tissue damage.

### 2.13 Actimeter

Six weeks-old C57Bl6 mice were randomly assigned to two groups according to peptide toxin treatment. The motor behavior was examined with an open field actimeter as previously described (Montnach et al., 2022). Briefly, mice were individually placed in an automated photocell activity chamber (Letica model LE 8811, Bioseb, France). Right after injection 0.9% NaCl (control) or 10 µg of peptide administration *via* intraperitoneally injection, the spontaneous motor activity was measured for 10 min using a movement analysis system (Bioseb, France). Similar experiments were repeated 60 min after injection.

### 2.14 Statistical analyses

Data are expressed as means ± standard error of the mean (S.E.M.) of *n* different experiments. The statistical comparison of values was carried out using *(i)* the parametric two-tailed Student’s t-test (either paired samples for comparison within a single population or unpaired samples for comparison between two independent populations), for *in vitro* experiments, or *(ii)* the one-way analysis of variance (ANOVA for comparison between the means of three or more independent populations) followed, if F was significant and if no variance inhomogeneity occurred, by *post hoc* pairwise *t*-tests with Dunnett’s correction, for *in vivo* experiments. Differences were considered to be statistically significant at *P* ≤ 0.05.

## 3 Results

### 3.1 Screening of *Cyriopagopus schmidti *venom on several hNa_v_ channel targets

**FIGURE 1:**
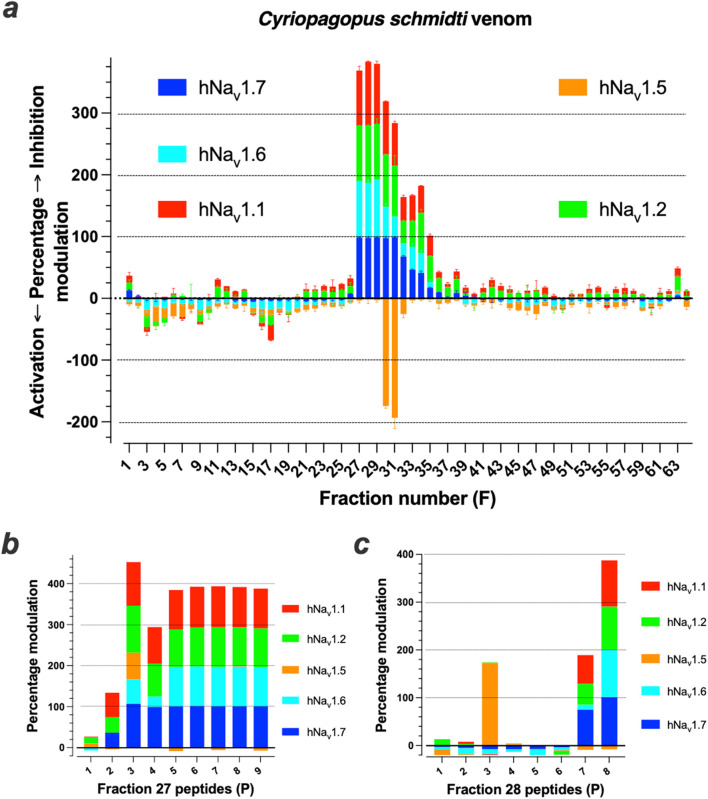
Screening of *Cyriopagopus schmidti* spider venom onto hNa_v_1.1, hNa_v_1.2, hNa_v_1.5, hNa_v_1.6 and hNa_v_1.7 isoforms. **(a)**, Screening results of primary RP-HPLC F1 to F64 onto the five hNa_v_ isoforms. **(b, c)**, Tests of nine and eight purified compounds from F27 **(b)** and F28 **(c)**, respectively, onto the five hNa_v_ isoforms. By convention, positive Y-axis values are Na^+^ influx inhibitors, while negative ones are Na^+^ influx activation.

The venom of the *C. schmidti* spider was fractionated into 64 fractions (F) (Supplementary Figure S2A). The bulk of material was mostly distributed between F21 and F32. After lyophilization and resuspension of the fraction into dH_2_O, the effects of the venom fractions were evaluated on the Na^+^ influx of five different hNa_v_ channel (hNa_v_1.1, hNa_v_1.2, hNa_v_1.5, hNa_v_1.6 and hNa_v_1.7), using the sodium-sensitive Asante Natrium Green-2 (ANG2) reporter dye. In these 96-well plate assays, performed in duplicate, cells coexpressing the hNa_v_ and inward rectifying Kir1.1 channels were incubated with veratridine for 1 h and challenged with a buffer containing high K^+^ concentration for cell depolarization. As shown, numerous fractions provided hNa_v_ channel modulation (Figure 1a). If an arbitrary threshold was set at 25% modulation, then 11 fractions out of 64 led to positive hits (17%). Ten fractions were found to modulate sodium influx through hNa_v_1.1 (9 inhibitors and one activator). Nine fractions were inhibitors of hNa_v_1.2. A total of four fractions are activators of hNa_v_1.5. Finally, seven and eight fractions are inhibitors of hNa_v_1.6 and hNa_v_1.7, respectively. None of the fractions inhibited hNa_v_1.5, while activating fractions of the other hNa_v_ channel subtypes were rare (a single exception for hNa_v_1.1, F17). Coherent with the fact that most peptides eluted in bulk within the central fractions, most active fractions were comprised between F27 and F35. F27 to F31 were those that had the highest blocking potencies on hNa_v_1.7 [between 97.5 ± 2.0 (n = 2) for F30 up to 98.5 ± 0.3 (n = 2) for F29]. In order to further select the fractions that contain the most potent peptides, we investigated the effect of highly diluted fractions on Na_v_-mediated Na^+^ influx (up to 1200-fold dilution) and found that F27 and F28 were those that kept maximal potencies (Supplementary Figure S2). These fractions had therefore the greatest likelihood to contain the most potent peptides. Therefore, we focused on these ones for further characterization, although of course the remaining fractions remain of interest to discover new compounds active on hNa_v_ channels. Mass spectrometry analyses by LC-ESI QTOF revealed the presence of at least eight and two major ions in F27 and F28, respectively (minor ions may be suppressed). The two major monoisotopic masses detected in F28 were 3,505.44 and 3,747.69 Da. Using cation exchange chromatography, we purified nine and eight subfractions, respectively, from F27 (Supplementary Figure S1B) and F28. MS analyses of these subfractions reveal the presence of eight original peptides (P) within F27 and six peptides within F28 (Supplementary Table S1). Two of the tested subfractions of F27 contain a mixture of two peptides, whereas no peptides could be detected in two subfractions of F28. Of note, F28P5 of 3,505.4 Da is similar to F27P4 and F28P6 of 3,747.7 Da is identical to F27P8, indicating that the total number of peptides within F27 and F28 was in fact 12 and not 14. Upon lyophilization, these peptides were resuspended at concentrations that should match the starting concentrations used for activity evaluation in Figure 1a. Among the nine subfractions from the cation exchange of fraction F27, eight of them displayed inhibitory activity onto at least three of the five hNa_v_ channels tested (Figure 1b). Subfraction 2 (F27P2) displayed limited inhibitory potency on hNa_v_1.1, hNa_v_1.2 and hNa_v_1.7, but spared hNa_v_1.5 and hNa_v_1.6. Subfraction 3 (F27P3) had potent pan-Na_v_ inhibitory effects, including on hNa_v_1.5, defining it as the sole peptide from this fraction partially inhibiting this channel type. Subfraction 4 (F27P4 mixed with F27P3) distinguished itself by a reduced potency on hNa_v_1.6. Finally, subfraction 5 (F27P5 mixed with F27P4), subfractions six to 9 (F27P6, F27P7 and F27P8) all displayed similar activities by blocking with high efficacies all the hNa_v_ channels tested, except hNa_v_1.5. Two subfractions from F28 (F28P7 and F28P8) showed inhibitory activity on at least three of the hNa_v_ channels tested (subfraction seven containing F28P5, and subfraction eight containing F28P6) with F28P6 being a general full inhibitor of hNa_v_1.1, hNa_v_1.2, hNa_v_1.6 and hNa_v_1.7, while F28P5 had a similar activity but spared hNa_v_1.6 (Figure 1c). F28P3 of 6,818.7 Da was a remarkable activator of hNa_v_1.5. Among these active peptides, we decided to *de novo* sequence five of them by a combination of Edman sequencing and MS/MS analyses: F27P3, F27P5, F27P7, F27P8 and F28P6.

### 3.2 Known and unknown huwentoxin peptides emerge from the screening and sequencing approach

**FIGURE 2:**
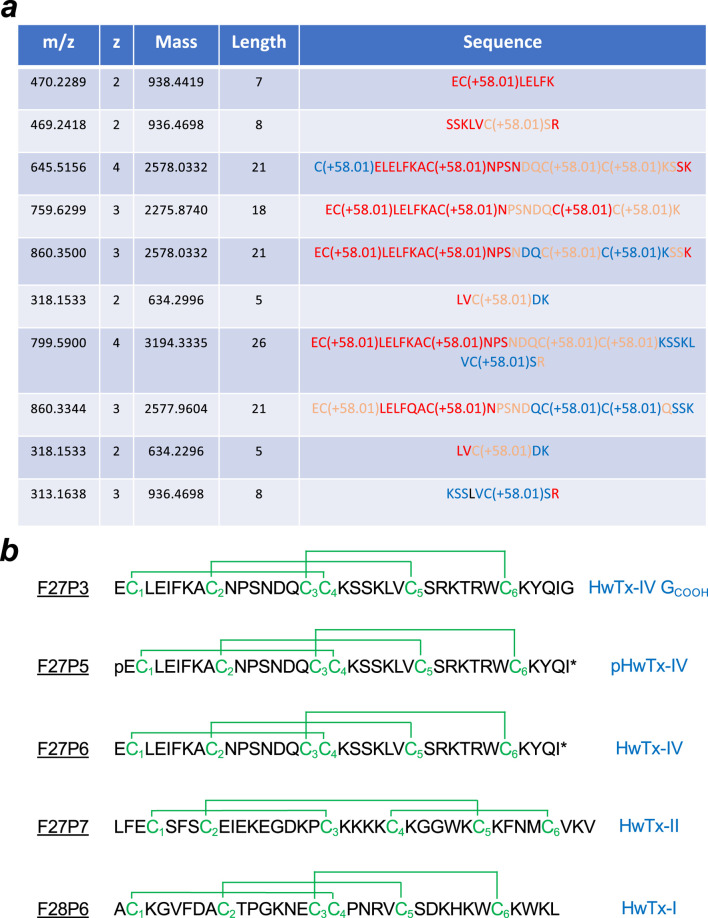
Amino acid sequencing of the five peptides regulating hNa_v_ channel activity. **(a)**, Partial sequences obtained from MS/MS data on F27P3 peptides following reduction, alkylation by iodoacetamic acid and trypsin digestion. The table has been built based on the results of *de novo* sequencing by the version 5.2 Peaks™ software (Bioinformatics Solutions Inc.) with the following settings: trypsin enzyme and carboxymethylation (C) as fixed modifications; mass accuracy on fragment ions at 0.1 Da; mass accuracy for the precursor mass at 10 ppm. Code color for the reliability index: red (>90%), salmon (80%–90%) and blue (60%–80%). **(b)**, Reconstructed sequences of the five peptides along with consensus disulfide bridging pattern. Known nomenclature is given right next to the sequences. Three out of four peptides were identified earlier. pHwTx-IV is a pyroglutamate at position one instead of glutamate.

The amino acid sequence of several of these peptides that possess pan-Na_v_ channel activity was determined. The chosen purified peptides had the following molecular weight as determined by LC-ESI QTOF MS: F27P3 (4161.9 Da), F27P5 (4085.9 Da), F27P6 (4103.9 Da), F27P7 (4281.0 Da) and F28P6 (3,747.7 Da same as F27P8) (Supplementary Table S1). For sequencing, Edman degradation was used in some cases and *de novo* MS/MS sequencing in all cases. First, all purified peptides were reduced using Tris (2-carboxyethyl) phosphine hydrochloride (TCEP) and the released thiol groups alkylated with iodoacetamide. The changes in molecular weights observed indicate that all four peptides possess six cysteine residues and hence three disulfide bridges in their native conformations. All four reduced/alkylated peptides were used as such for sequence determination. For LC-ESI-MS/MS *de novo* sequencing the reduced/alkylated peptides were digested first by either trypsin or V8 protease before undergoing analyses. Partial sequences resulting from these MS/MS analyses are shown for F27P3 (Figure 2a), and reconstructed sequences for all five peptides are shown (Figure 2b). The results of this screening were consistent with earlier findings on this venom. F28P6 turned out to be huwentoxin-I (HwTx-I) that belongs to Na_v_-targeting spider toxins (NaSpTx-1) family 1 (Klint et al., 2012). This toxin has 33 amino acid residues in length, is carboxylated at the C-terminus and displays the typical Inhibitor Cystine Knot (ICK) fold. It was characterized initially for its activity on N-type voltage-gated Ca^2+^ channels (Peng et al., 2001), but recently also recognized for its activity on Na_v_ channels (Wang et al., 2012). On Na_v_ channels, it seems to block TTX-S currents of rat DRG neurons and hippocampal neurons. This toxin has shown promising antinociceptive results (Chen et al., 2005; Che et al., 2009) in spite of signs of neurotoxicity as well (Liang et al., 1993; Zhou et al., 1997). Recently, we characterized synthetic HwTx-I as a potent blocker of hNa_v_1.7 with an IC_50_ of 25 nM (Nicolas et al., 2019). F27P7 is also a known peptide and shares 100% sequence identity with HwTx-II. This peptide of 37 amino-acids adopts a novel and unique disulfide bridge organization, different from the classical ICK fold (Shu et al., 2001; Shu et al., 2002) and comes with a C1-C3, C2-C5 and C4-C6 bridging pattern (Figure 2B). Curiously, besides its insecticidal activity, the chemical synthesis of the peptide was not reported. In addition, our data represent the first report of an activity on Na_v_ channels. This peptide is thus another interesting candidate for chemical synthesis and in-depth investigation of its activity on Na_v_ channel isoforms. F27P6 is a rediscovery of HwTx-IV, a 35 amino acid peptide, again with an ICK fold and a characteristic C-terminal amidation. It belongs to the NaSpTx-1 family of spider peptides. This peptide has been extensively studied in the past for its antinociceptive properties (Liu et al., 2014), but its inhibitory toxic activity against Na_v_1.6 prevented further development for pain treatment (Goncalves et al., 2018), although later SAR investigation proved that this toxic activity could be minimized (Lopez et al., 2021). F27P5 is a pyroglutamate version of HwTx-IV, another rediscovery in this venom as it was disclosed earlier (Rong et al., 2013). Finally, F27P3, that differs from F27P6 by an additional mass of 58 Da, turned out to be another close analogue of HwTx-IV (Figure 2B). As a matter of fact, F27P3 has exactly the same amino acid sequence and disulfide bridging pattern than HxTx-IV, except that it possesses an additional C-terminal residue and that the amidation is lacking. This HwTx-IV analogue was termed nHwTx-IV G_COOH_ (n for natural) in the remaining of the description. Earlier reports on HwTx-IV SAR had shown that the loss of amidation produced a severe reduction in peptide potency for blocking hNa_v_1.7 expressed in cell lines (over 50-fold) (Minassian et al., 2013; Sermadiras et al., 2013; Lopez et al., 2021). The addition of a Gly residue (position 36) was reported to improve slightly the potency without matching that of the native HwTx-IV (Sermadiras et al., 2013), but the situation was significantly improved by either an additional C-terminal amidation or a C-terminal Lys residue at either position 36 in replacement of Gly36 (Lopez et al., 2021) or 37 after Gly36 (Minassian et al., 2013). Besides these changes in potency onto hNa_v_1.7, altering the C-terminal sequence of HwTx-IV was found to change the selectivity ratio for different Na_v_ isoforms as well (Minassian et al., 2013; Neff et al., 2020; Lopez et al., 2021) indicating that nHwTx-IV G_COOH_, present in the venom of the *C. schmidti* spider, has potentially greater interest for modulating the physiology of envenomated animals than initially thought.

### 3.3 Chemical synthesis and *in vitro* folding of synthetic (s)HwTx-IV G_COOH_

**FIGURE 3:**
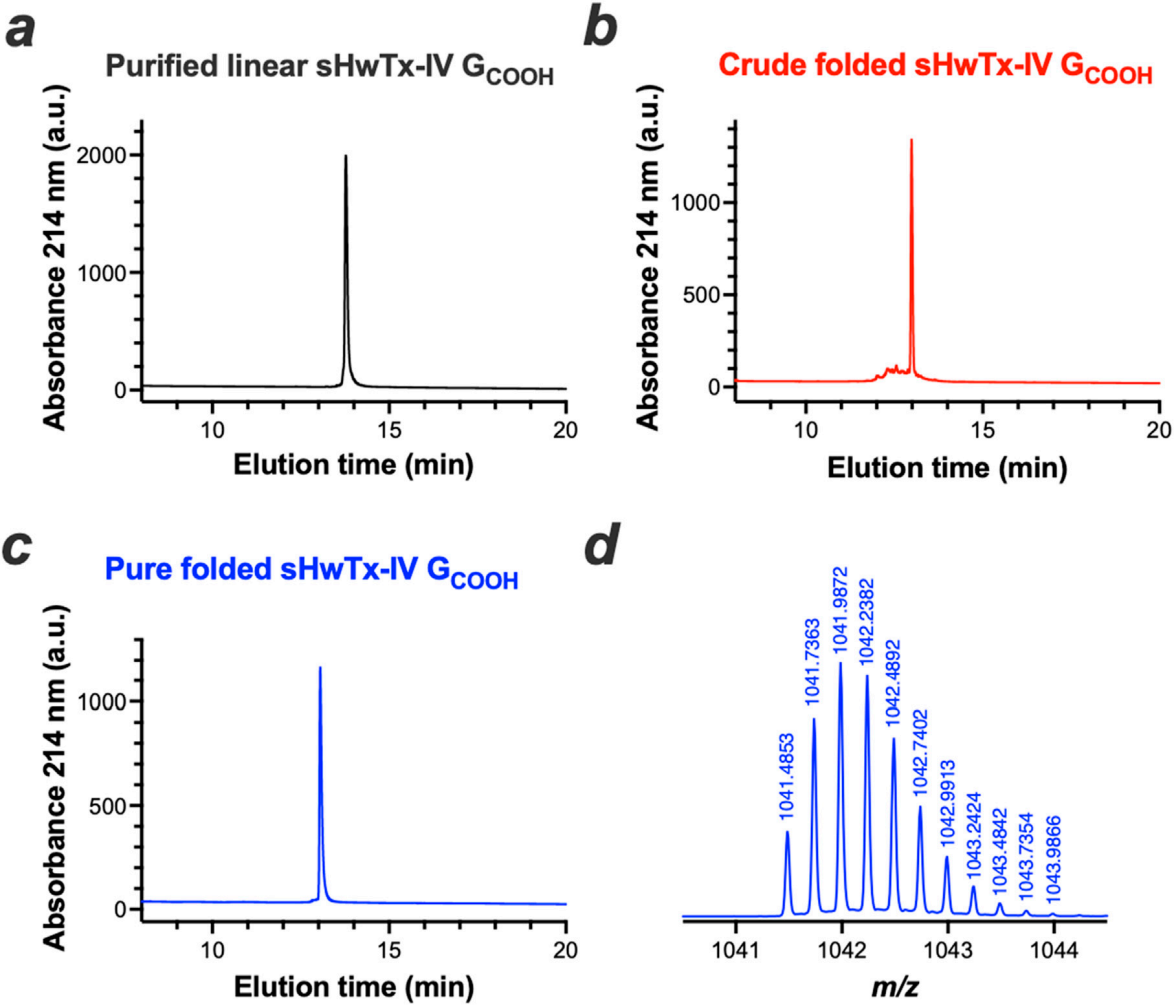
Chemical synthesis of sHwTx-IV G_COOH_. **(a-c)**, RP-HPLC elution profiles of purified linear **(a)**, crude folded/oxidized **(b)** and pure folded/oxidized sHwTx-IV G_COOH_ **(c, d)**, [M+4H]^4+^ mass spectrum of purified folded/oxidized sHwTx-IV G_COOH_.

To get a better understanding of the activity of this novel peptide, it was chemically synthesized, folded/oxidized and purified to homogeneity (Figure 3). The MS spectrum of sHwTx-IV G_COOH_ confirmed proper chemical synthesis of the compound with a monoisotopic mass of [M+4H]^4+^ = 1,041.4853 m*/z* corresponding to a molecular mass of 4161.94 Da that is identical to nHwTx-IV G_COOH_.

### 3.4 Potency of nHwTx-IV G_COOH_ and sHwTx-IV G_COOH_ on Na_v_ channels

**FIGURE 4:**
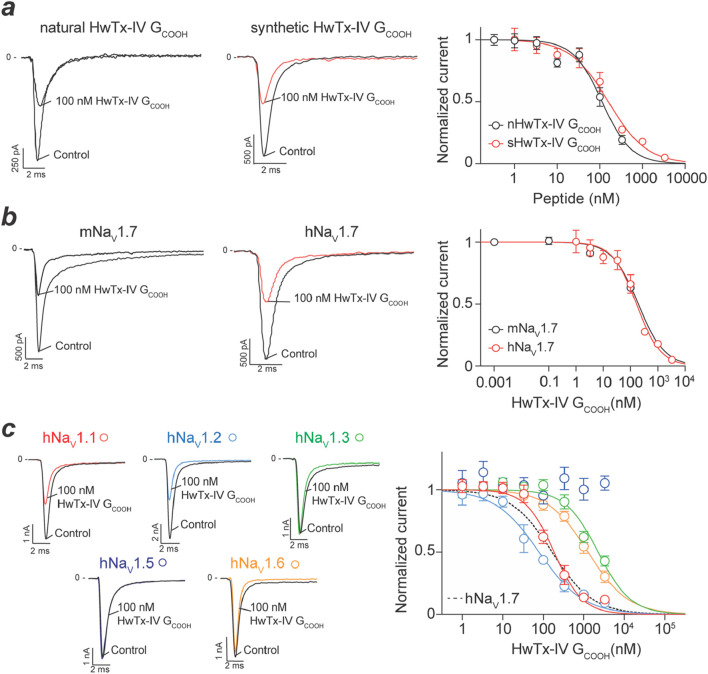
Inhibitory activity of natural and synthetic HwTx-IV G_COOH_ on various Na_v_ isoforms expressed in cell lines. **(a)**, Concentration-response effects of natural (n) and synthetic (s) HwTx-IV G_COOH_ on Na^+^ currents carried by hNa_v_1.7 channels. Left panel, trace examples at 100 nM peptide concentration. Right panel, dose-response curves providing IC_50_ values of 113 nM (nHwTx-IV G_COOH,_ 95CI (86.9–144.6) and n_H_ = −1.28) and 158 nM (sHwTx-IV G_COOH_, 95CI (115.5–218.1) and n_H_ = −0.97). **(b)**, Comparison of the potency of sHwTx-IV G_COOH_ on mNa_v_1.7 and hNa_v_1.7 illustrating similarity. IC_50_ value of 193 nM on mNa_v_1.7 (95CI (68.72–582.6) and n_H_ = −0.90). **(c)**, Dose-response curves illustrating the potency of sHwTx-IV G_COOH_ onto several hNa_v_ isoforms. Left panel, representative trace examples of the effect of 100 nM sHwTx-IV G_COOH_. Right panel, dose-response curves. IC_50_ values are: 185 nM (hNa_v_1.1), 79 nM (hNa_v_1.2), 2,260 nM (hNa_v_1.3), 1,268 nM (hNa_v_1.6) and 165 nM (hNa_v_1.7, 95CI (124.8–218.4) and n_H_ = −0.98).

An advantage of automated patch-clamp technology is that the experimental tests require very little material quantities. Therefore, purified peptides can be tested for their potency on a given channel target allowing direct comparison with the potency of the synthetic counterpart. We took this opportunity to test nHwTx-IV G_COOH_ onto the hNa_v_1.7 target stably expressed in CHO cells. As shown, nHwTx-IV G_COOH_ potently inhibited hNa_v_1.7 currents with an IC_50_ value of 113 nM (Figure 4a). Importantly, a similar potency of 158 nM was recorded for sHwTx-IV G_COOH_, validating the proper synthesis of this analogue. These potency values are of the same order as the reported IC_50_ value of HwTx-IV without amidation (reported IC_50_ value of 176 nM) (Lopez et al., 2021) but lacking this Gly^36^ residue. Next, we evaluated the potency of sHwTx-IV G_COOH_ for inhibiting mNa_v_1.7, as on several occasions we noticed species differences in natural peptide potencies. As shown, sHwTx-IV G_COOH_ inhibited mNa_v_1.7 (IC_50_ = 193.3 nM) with a potency that closely resembles the one obtained on hNa_v_1.7 (Figure 4b), indicating that the peptide could be properly evaluated on mNa_v_1.7 from mouse DRG neurons. Finally, we examined the Na_v_ channel selectivity of sHwTx-IV G_COOH_ (Figure 4c). The peptide inhibited the various Na_v_ isoforms in the following rank order of potencies: hNa_v_1.2 (IC_50_ = 79 nM) > hNa_v_1.7 (IC_50_ = 165 nM) > hNa_v_1.1 (IC_50_ = 185 nM) >> hNa_v_1.6 (IC_50_ = 1,268 nM) > hNa_v_1.3 (IC_50_ = 2,260 nM) >> hNa_v_1.5 (no inhibition up to 3,300 nM). The partial blocking effect observed on hNa_v_1.5 Na^+^ influx mediated by F27P3 (nHwTx-IV G_COOH_) (Figure 1b) was therefore not confirmed. However, both tests differed since veratridine was present in the Na^+^ influx test which may uncover a hidden binding site. hNa_v_1.4 was also found to be not affected by sHwTx-IV G_COOH_, which is coherent with the effects of HwTx-IV. Therefore, we conclude that sHwTx-IV G_COOH_ was mostly active on three Na_v_ isoforms (hNa_v_1.1, hNa_v_1.2 and hNa_v_1.7) at concentrations below 1 μM. With a potency of 158 nM on hNa_v_1.7, sHwTx-IV G_COOH_ was therefore 16-fold less potent than HwTx-IV itself on this target (Lopez et al., 2021). Next, we evaluated the potency of sHwTx-IV G_COOH_ on Na_v_ currents from mouse DRG neurons to examine the coherence of these findings.

### 3.5 Effects of sHwTx-IV G_COOH_, compared to HwTx-IV, on TTX-S and TTX-resistant (TTX-R) Na^+^ currents from adult mouse DRG neurons

**FIGURE 5:**
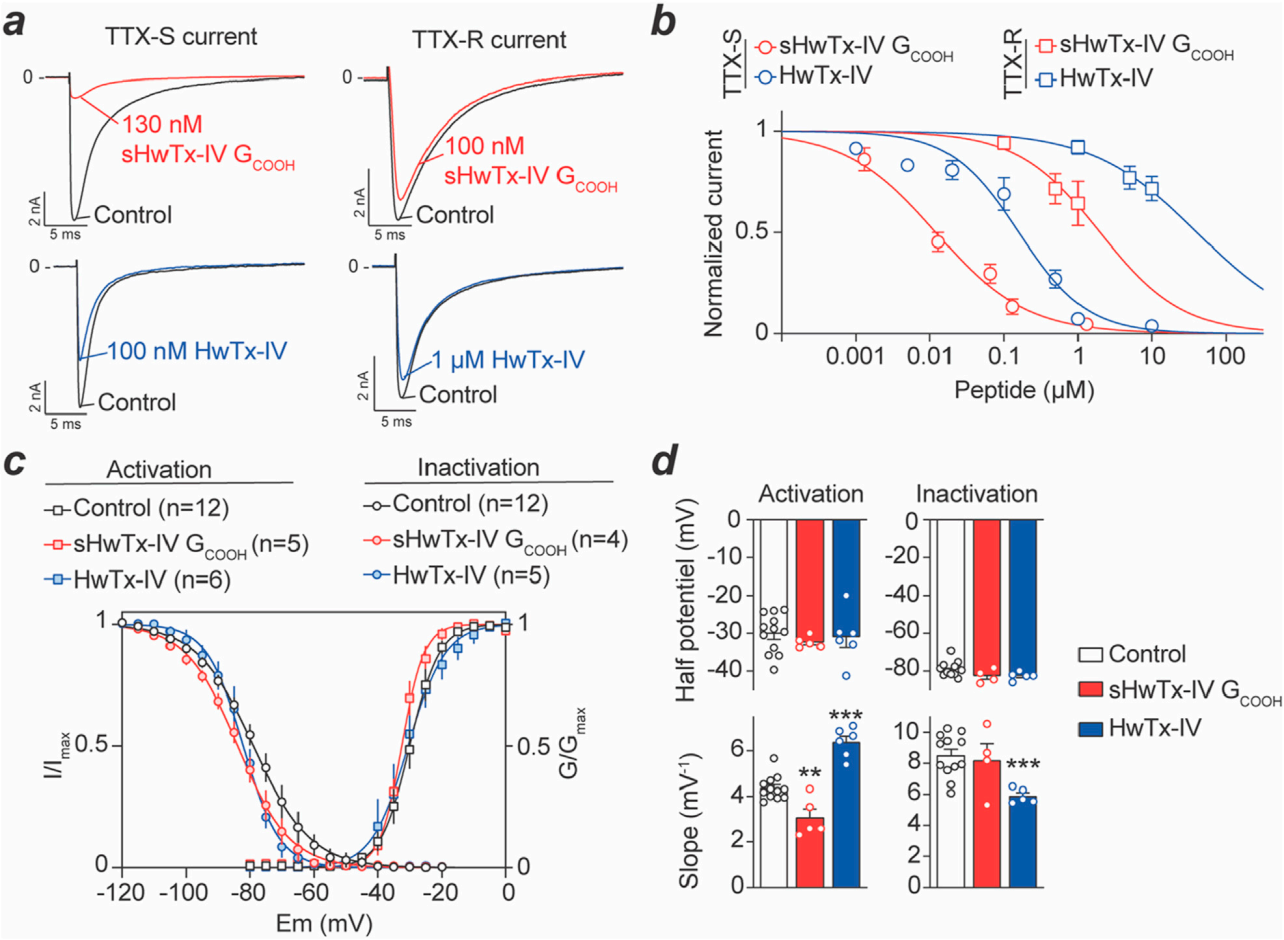
Concentration-response curve of sHwTx-IV G_COOH_ and HwTx-IV on mouse DRG TTX-S and TTX-R Na^+^ currents. **(a)**, Representative current traces measured during 50-m test-pulses to −20 mV from a holding potential of −100 mV before and after application of one of the two peptides at 100 nM, 130 nM or 1 μM onto TTX-S and TTX-R Na^+^ currents. **(b)**, Average dose-response curves for both peptides on the two types of Na^+^ currents (TTX-S and TTX-R). Each value is expressed as a percentage of the current reached before peptide application and represents the mean ± S.E.M. from 3 to 14 DRG neurons. Theoretical curves are calculated according to the Hill equation with IC_50_ and n_H_ values of 14 nM and 0.7 (*r* ^2^ = 0.982) and 2 μM and 0.8 (*r* ^2^ = 0.976) for sHwTx-IV G_COOH_ on TTX-S and TTX-R currents, respectively, and 133 nM and 0.8 (*r* ^2^ = 0.927) and 28 μM and 0.8 (*r* ^2^ = 0.986) for HwTx-IV effects on TTX-S and TTX-R currents, respectively. **(c,d)**, Effects of sHwTx-IV G_COOH_ and HwTx-IV on voltage-dependence of activation and inactivation of the TTX-S current. Steady-state inactivation- (circles) and conductance- (squares) voltage relationships, before (black circles) and after exposure to either 13 nM sHwTx-IV G_COOH_ (red) or 100 nM HwTx-IV (blue). Each value represents the mean ± S.E.M. of data obtained from 6 to 12 DRG neurons, and is expressed as percentage of either maximal peak amplitude of current at strongly negative pre-pulse voltages (left) or maximal conductance calculated at strongly positive test-pulse voltages (right). **: *P* < 0.01 and ***: *P* < 0.001 versus control.

Na^+^ currents from two types of DRG neurons could be recorded that differed by their TTX sensitivity. 76% of DRG neurons (*i.e.* 75 of a total of 99 cells recorded) had average cell diameters of 26.5 ± 0.7 µm and displayed essentially TTX-S current. In these neurons, the Na^+^ current was decreased by 100 nM TTX to 9.5% ± 1.7% of initial peak amplitude values within 90 s. This is coherent with earlier reports (Djouhri et al., 2003; Black et al., 2012). Also, 10 nM protoxin-II, which is Na_v_1.7 selective to a large extent (Montnach et al., 2021), inhibits 80% of the Na^+^ current carried by these TTX-sensitive neurons (Supplementary Figure S3), a result coherent with previous reports (Alexandrou et al., 2016; Flinspach et al., 2017). 24% of the remaining neurons *(i.e.* 24 over 99 cells recorded) had significantly larger cell diameters of 34.2 ± 1.6 µm (*P* < 0.001) and displayed both TTX-S and TTX-R components. Indeed, the Na^+^ current was decreased to 64.8% ± 4.2% of initial peak amplitude by 100 nM TTX, also coherent with the presence of Na_v_1.8 in larger neurons (Ramachandra et al., 2013). The effects of both HwTx-IV and sHwTx-IV G_COOH_ were tested on these two subsets of DRG neurons. For TTX-S neurons, the Na^+^ currents were recorded in the absence of TTX, while for TTX-R neurons, the cell medium was supplemented with 100 nM TTX to ensure the recording of pure TTX-R Na^+^ currents. Interestingly, representative current traces illustrate that 130 nM sHwTx-IV G_COOH_ potently blocked TTX-S Na^+^ currents from DRG neurons, while largely sparring TTX-R Na^+^ currents (Figure 5a). At this concentration, sHwTx-IV G_COOH_ was far more potent than HwTx-IV itself on TTX-S currents which was unexpected from the findings on mNa_v_1.7 expressed in cell lines (Figure 4b) that would predict milder potency. Dose-response curves were built to further investigate the relative potencies of both peptides on these TTX-S and TTX-R currents (Figure 5b). As shown, sHwTx-IV G_COOH_ potently blocked TTX-S DRG currents with an IC_50_ value of 14 nM, which was 11.8-fold more potent than on mNa_v_1.7 currents. Interestingly, HwTx-IV itself inhibited TTX-S currents with an IC_50_ of 133 nM, which was conversely 13.4-fold less potent than on hNa_v_1.7 (Lopez et al., 2021). Overall, on mouse DRG TTX-S Na^+^ currents, vastly supported by Na_v_1.7, sHwTx-IV G_COOH_ was thus 9.5-fold more potent than HwTx-IV itself, which is therefore the exact opposite finding than on stable cell lines expressing hNa_v_1.7. These data question the validity of stable cell lines for the identification of the best performing compounds active on Na_v_1.7 channels. The recent finding of a plant toxin, excelsatoxin, active on Na_v_1.7 from DRG neurons but not from stable cell lines provides support to this questioning (Jami et al., 2023). The lack of potency of both HwTx-IV and sHwTx-IV G_COOH_ on TTX-R Na^+^ currents was confirmed with calculated IC_50_ values of approximately two and 28 μM, respectively.

Concerning the kinetics of block by these two peptides, we report only minor differences. Indeed, 130 nM sHwTx-IV G_COOH_ reached stationary TTX-S peak current amplitude block within 2.6 ± 0.6 min (n = 8), whereas 1 μM HwTx-IV, that produced equivalent block, reaches this stationary level in 2.7 ± 0.2 min (n = 7). Noteworthy, under our experimental conditions, the effects of both sHwTx-IV G_COOH_ and HwTx-IV on TTX-S current were reversible. In particular, TTX-S peak current amplitude following wash-out of 130 nM sHwTx-IV G_COOH_ for 13 ± 1 min was not very different (*P* = 0.555) from that recorded before peptide application (*i.e.*, −3.2 ± 0.7 nA and −3.6 ± 0.5 nA (n = 12), respectively). The same observation was made following wash-out of 1 μM HwTx-IV for 10 ± 2 min with current amplitude levels not significantly different (*P* = 0.749) from that recorded before peptide application (*i.e.*, −4.3 ± 0.6 nA and −4.8 ± 0.7 nA (n = 7), respectively).

### 3.6 DRG TTX-S Na^+^ biophysical parameters affected by sHwTx-IV G_COOH_

Both sHwTx-IV G_COOH_ (1.3–65 nM) and HwTx-IV (5–500 nM), at concentrations that did not fully block TTX-S Na^+^ currents, did not alter activation or inactivation kinetics. Indeed, the activation time (Time_A_), corresponding to a 10%–90% current increase, and the inactivation time (Time_I_), measured by a 90% to 10% current decrease, did not significantly (*P* ≥ 0.081) differ in the absence and presence of one or other of these peptides (Table 1). Similarly, we analyzed the effects of the peptides on the voltage-dependence of both activation and inactivation for TTX-S Na^+^ currents (Supplementary Figure S4). At intermediate blocking concentrations, sHwTx-IV G_COOH_ (13 nM) or HwTx-IV (100 nM) did not markedly affect the voltage-dependencies of the remaining currents as witnessed by the absence of noticeable shifts in activation-voltage and steady-state inactivation-voltage relationships (Figures 5c, d). Indeed, both the voltage corresponding to 50% maximal conductance (V_A50%_) and the voltage at which 50% of the channels were inactivated (V_I50%_) are not significantly (*P* ≥ 0.064) different in the absence and in the presence of either sHwTx-IV G_COOH_ or HwTx-IV. V_A50%_ control was −30 ± 1 mV (n = 12) and was not modified by either HwTx-IV (−31 ± 3 mV, n = 6) or sHwTx-IV G_COOH_ (−32 ± 1 mV, n = 5). Similarly, V_I50%_ control was −79 ± 4 mV (n = 12) and not significantly modified by HwTx-IV (−83 ± 2 mV, n = 5) or sHwTx-IV G_COOH_ (−82 ± 2 mV, n = 4). However, slight, but significant (*P* < 0.002), changes occurred in the slope (k_H_) of steady-state inactivation-voltage curves in the presence of HwTx-IV and in the slope (k_G_) of conductance-voltage curves in the presence of either sHwTx-IV G_COOH_ or HwTx-IV, compared to those in the absence of peptides. One possibility remains that these peptides affect the voltage-dependence of activation of peptide-bound channels to a level that goes beyond possible current measurements (Xiao et al., 2008).

**TABLE 1:** Time (Time_A_) of current increase from 10% to 90% (activation) and that (Time_I_) of current decrease from 90% to 10% (inactivation). Each value represents the mean ± S.E.M. from *n* DRG neurons, as indicated.

Peptide concentration	Time_A_ (ms)		Time_I_ (ms)	
	Control	sHwTx-IV G_COOH_	Control	sHwTx-IV G_COOH_
1.3 nM (n = 10)	0.27 ± 0.07	0.31 ± 0.06	6.94 ± 1.35	6.93 ± 1.23
13 nM (n = 14)	0.26 ± 0.13	0.27 ± 0.07	7.08 ± 1.40	6.66 ± 1.42
65 nM (n = 8)	0.27 ± 0.09	0.28 ± 0.11	6.86 ± 0.67	6.59 ± 1.24

	Control	HwTx-IV	Control	HwTx-IV
5 nM (n = 6)	0.30 ± 0.07	0.28 ± 0.05	6.83 ± 1.46	6.82 ± 0.69
100 nM (n = 6)	0.27 ± 0.04	0.34 ± 0.07	6.95 ± 0.76	6.50 ± 1.49
500 nM (n = 3)	0.28 ± 0.05	0.29 ± 0.04	7.03 ± 0.42	6.76 ± 0.70

### 3.7 Effects of sHwTx-IV G_COOH_, compared to HwTx-IV, on pain sensitivity of mice *in vivo*

**FIGURE 6:**
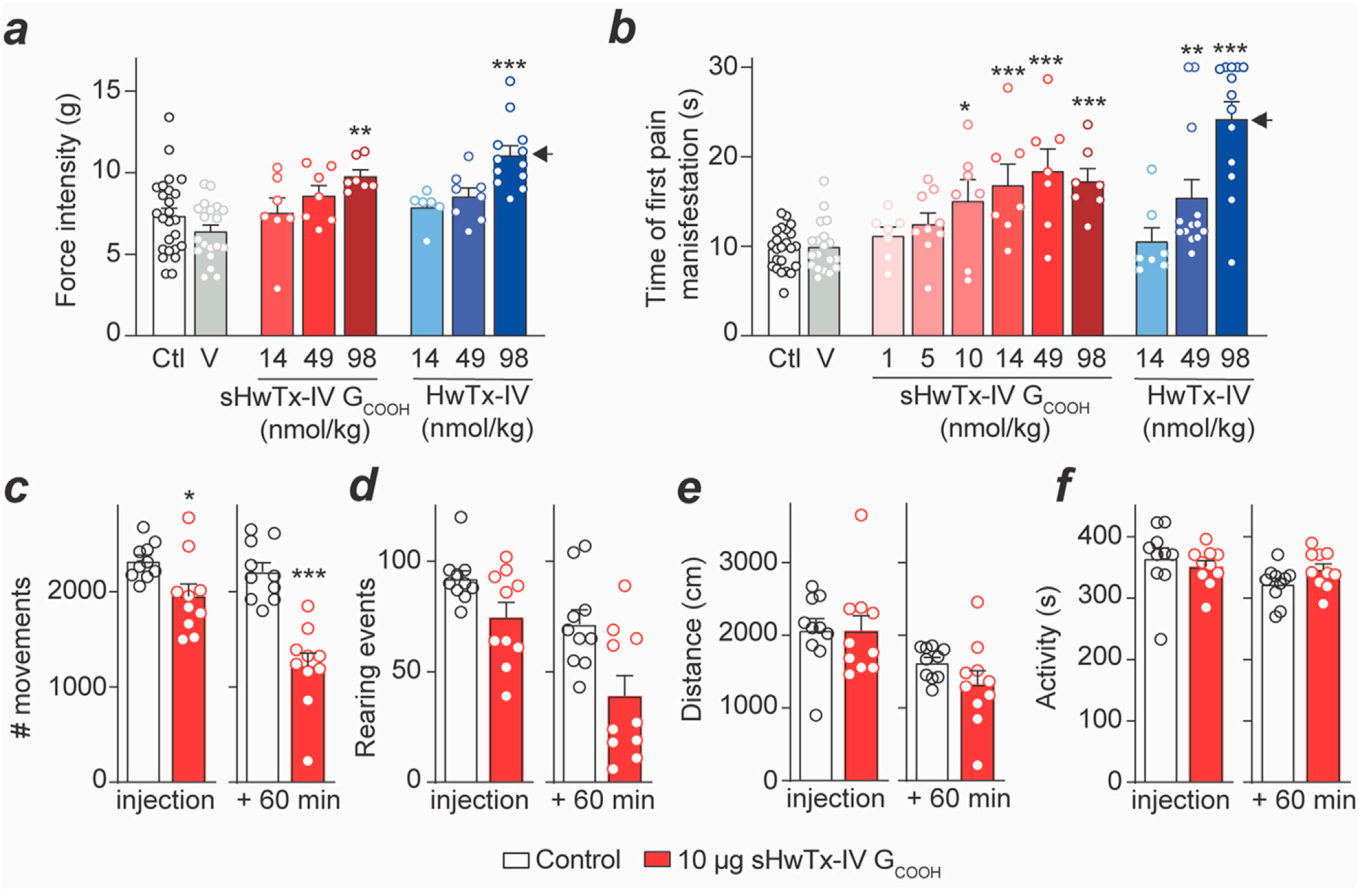
Effects of sHwTx-IV G_COOH_ and HwTx-IV on tactile and heat sensitivity of mice *in vivo*. **(a)**, Tactile sensitivity of mice was assessed using the automated plantar von Frey apparatus, by determining the force intensity at which the animals removed their hind limb submitted to an increasing fibre pressure. The same mice were tested before (no injection) and after intraplantar injection of vehicle (maximum 15 µL of PBS +0.1% BSA), sHwTx-IV G_COOH_ (14–98 nmol/kg) or HwTx-IV (14–98 nmol/kg). **(b)**, The heat sensitivity of mice was assessed using a hot-plate set at 55.0°C ± 0.2°C, by determining the latency for the animals to shake the right hind limb. Ten groups of minimum six mice were tested after intraplantar injection of vehicle, sHwTx-IV G_COOH_ (1–98 nmol/kg) or HwTx-IV (14–98 nmol/kg), while an 11th group of animals was tested without any injection. *: *P* < 0.05, **: *P* < 0.01 and ***: *P* < 0.001 versus vehicle-injected mice. The black arrow indicates motor impairment of all injected mice. Peptide concentration injected was equivalent to 3 μM (1 nmol/kg) up to 294 μM (98 nmol/kg). **(c)**, Number of mice movements during the first 10 min after intraperitoneal injection of 10 μg sHwTx-IV G_COOH_ injection or after 60 min compared to control. *: *P* < 0.05 and ***: *P* < 0.001 versus vehicle-injected mice. **(d)**, Number of rearing events immediately after and 60 min after intraperitoneal injection of 10 μg sHwTx-IV G_COOH_. **(e)**, Distance travelled by mice for the two times post-injection of 10 μg sHwTx-IV G_COOH_. **(f)**, Activity duration of the mice for the two times post-injection of 10 μg sHwTx-IV G_COOH_.

The antinociceptive effects of sHwTx-IV G_COOH_ (14–98 nmol/kg) compared to HwTx-IV (14–98 nmol/kg) were first evaluated on the automated von Frey assay. Tactile sensitivity testing illustrates that the force intensity at which mice, injected with 98 nmol/kg of sHwTx-IV G_COOH_ or HwTx-IV, removed their hind limb in response to fibre pressure, significantly (*P* < 0.010) increased, compared to mice injected with the vehicle (Figure 6a). For this particular test, no dose efficiency difference could be observed between both peptides. Next, the hot-plate assay was used to evaluate the effects of sHwTx-IV G_COOH_ (1–98 nmol/kg), compared to those of HwTx-IV (14–98 nmol/kg), on mice pain sensitivity. A significant (*P* < 0.050) increase in the reaction time to heat, *i.e.*, the latency to shake the right hind limb, was observed for mice injected with 10–98 nmol/kg of sHwTx-IV G_COOH_ or 49–98 nmol/kg of HwTx-IV, compared to mice injected with the vehicle (Figure 6b). In this test, however, sHwTx-IV G_COOH_ was clearly analgesic at lower doses compared to HwTx-IV (threshold at 10 nmol/kg versus 49 nmol/kg). Noteworthy, the injection itself was innocuous since no significant difference (*P* > 0.148) in the force intensity or reaction time to heat occurred between mice injected with the vehicle and non-injected animals (Figure 6).

During this study, we failed to detect toxicity of sHwTx-IV G_COOH_ at the highest concentrations used for limb injection (no abnormal mice behavior and absence of death including 24 h later). In contrast, intraplantar injections of 98 nmol/kg of HwTx-IV caused evident motor impairment within 30–45 min in all injected animals, which is likely to explain the high force intensity and long reaction time to heat observed for these animals (Figure 6). The high toxicity of HwTx-IV is also evident from the death observed in 31% of animals, all occurring within 60 min post-injection. 98 nmol/kg corresponds approximately to an intraplantar injection of about 12 μg HwTx-IV. Of course, death is not induced by the peptide presence in the limb but rather by its passive diffusion outside the limb in the systemic circulation, thus at much lower peptide concentrations. To further assess the potential toxicity of sHwTx-IV G_CCOH_, we performed an actimeter study on mice using an equivalent concentration in the body (faster systemic administration). As shown, at this concentration, we observe a reduction in the number of movements post-injection that is accentuated 60 min later (Figure 6c). Rearing was reduced also (Figure 6d). In contrast, both the distance traveled by the mice (Figure 6e) and the duration of activity (Figure 6f) remained unaffected by sHwTx-IV G_CCOH_. Seven out of 10 animals had signs of hindlimb weaknesses. There were no signs of suffering and none of the animals died. These results indicate that sHwTx-IV G_COOH_ induces less *in vivo* toxicity than HwTx-IV with the benefit of a preserved analgesia (see also (Goncalves et al., 2018)).

## 4 Discussion

In the course of this screening project, we identified one previously undetected analog of HwTx-IV, which is characterized by an extra C-terminal Gly residue and the absence of C-terminal amidation. A second analogue was confirmed which is pHwTx-IV that differs from HwTx-IV by pyroglutamation. Our ability to detect three closely related analogues of HwTx-IV, with minor differences in sequence, within the course of the venom fractionation and the screening process, informs about the robustness of the screening procedure used herein. The existence of a mature HwTx-IV peptide with an additional Gly residue displaying a C-terminal carboxylation in the venom of *C. schmidti* interrogates on the function of this Gly residue. It likely represents a reminiscent fraction of an incompletely processed precursor form of HwTx-IV itself, although we cannot rule out that it stands as a novel isoform of the peptide by itself. Arguments are manyfold for HwTx-IV G_COOH_ representing an incompletely processed HwTx-IV peptide. Indeed, peptidyl-glycine α-amidating monooxygenases (PAM) are bifunctional enzymes with peptidylglycine α-hydroxylating monooxygenase and peptidyl-α-hydroxyglycine α-amidating lyase activities that remove the Gly residue and amidate the preceding amino acid. The fact that a carboxylated form of HwTx-IV could not be identified in the venom of *C. schmidti* indicates that such an enzyme is probably at work in the venom gland for the production of HwTx-IV. PAMs are present in secretory granules (Oyarce and Eipper, 1995) and contribute to the maturation of oxytocin, vasopressin, calcitonin and substance P. Since HwTx-IV G_COOH_ is detected in the venom fractions, the maturation process is probably incomplete in the venom gland. PAMs have been involved in the maturation of other natural peptides as well. Indeed, ω-conotoxin MVIIA was shown to be synthesized as a precursor peptide also containing a C-terminal glycine residue. This C-terminal Gly is also post-translationally processed by oxidative cleavage but its presence was shown to facilitate the folding efficiency of ω-conotoxin MVIIA (Price-Carter et al., 1996). Whether HwTx-IV G_COOH_ folds better than HwTx-IV was not determined in our hands. It is worth noting however that HwTx-IV is not the most mature form of this peptide, since pHwTx-IV underwent an additional post-translational processing under the form of pyroglutamation.

The activity of PAMs is believed to be essential as amidated peptides are considered to be more stable since amidation prevents peptide degradation and prolongs half-life. Also, amidated peptides have often higher receptor affinity and enhanced physiological effects (Eipper et al., 1992a; Eipper et al., 1992b). The case of ω-conotoxin MVIIA is exemplary with its 10-fold better affinity for N-type calcium channels than the non-amidated ω-conotoxin MVIIA-Gly-COOH (Price-Carter et al., 1996). In fact, the same hold true for HwTx-IV in its amidated form since it is over 10-fold more potent than HwTx-IV G_COOH_ on Na_v_1.7 channels expressed in cell lines. However, this finding hides interesting features that were not considered in sufficient details in earlier reports. First, HwTx-IV G_COOH_ is three times more potent on hNa_v_1.7 than carboxylated HwTx-IV (lacking amidation) (Sermadiras et al., 2013), indicating that the extra Gly residue adds *per se* a form of competitive advantage. Second, it affects the selectivity profile of the peptide which is coherent with the importance of the C-terminal sequence as a pharmacophore (Lopez et al., 2021). Third, HwTx-IV G_COOH_ is far more potent on mouse DRG Na_v_1.7 indicating that the pharmacology of this native channel is largely influenced by unknown factors not present in cell lines. While Na^+^ channel β subunits are evident candidates, more elusive proteins may be at work as well. Recently, TMEM233, a member of the dispanin family expressed in mouse DRG neurons, was shown to be critical for the modulation of Na_v_1.7 channel activity by excelsatoxin A, a pain-causing knottin peptide from the *Dendrocnide excelsa* tree (Jami et al., 2023). Whether TMEM233 or other members of the dispanins may regulate the activity of HwTx-IV and its analogues would be an interesting question to pursue. Our findings indicate that this regulation would influence HwTx-IV and HwTx-IV G_COOH_ pharmacology in opposite directions by degrading HwTx-IV potency and conversely improving the one of HwTx-IV G_COOH_. However, other explanations cannot be ruled out such as the existence of a splice variant in DRG neurons, not matching the one of the stable cell line, or differences in dipole moment or membrane lipid compositions between HEK293 cells and DRG neurons.

Noteworthy, the improved potency of HwTx-IV G_COOH_ on mouse DRG Na_v_1.7-mediated currents is paralleled by an improved antinociceptive potency *in vivo*. The benefit was more obvious on thermal than on tactile pain. This finding is however a clear indication that peptide potency on DRG Na^+^ currents is a better signal for detecting the antinociceptive potential of the compound than potency on a Na_v_ channel expressed in a cell line. Also, HwTx-IV G_COOH_ was found to be less toxic than HwTx-IV in mice (motor impairment or death), which is in agreement with an even lower IC_50_ value for hNa_v_1.6 inhibition, the channel isoform that controls neuromuscular transmission (1,268 nM (Figure 4c) versus 226 nM (Lopez et al., 2021) for HwTx-IV under similar recording conditions). This is also another important step for the use of HwTx-IV G_COOH_ as a safe antinociceptive agent.

## Data Availability

The original contributions presented in the study are included in the article/Supplementary Material, further inquiries can be directed to the corresponding author.
