# Supplementary material for: The venom of Cyriopagopus schmidti spider contains a natural huwentoxin-IV analogue with unexpected improved analgesic potential
Source: Front Pharmacol. 2025 Apr 10;16:1566312. doi: 10.3389/fphar.2025.1566312 (PMC12019880; doi:10.3389/fphar.2025.1566312)
Supplement: Supplementary file 1 [file DataSheet1.docx]

Supplementary Material

The venom of *Cyriopagopus schmidti* spider contains a natural huwentoxin-IV analogue with unexpected improved analgesic potential

Aurélie Antunes^1,2,3^, Jérôme Montnach^3^, Kuldip Khakh^4^, Ludivine Lopez^3^, Baptiste Thomas^2^, Barbara Ribeiro Oliveira-Mendes^3^, Lucie Jaquillard^2^, Denis Servent^1^, Rémy Béroud^2^, Charles J. Cohen^4^, Evelyne Benoit^1^ and Michel De Waard^2,3,5,^*

# Supplementary Figures and Tables

**
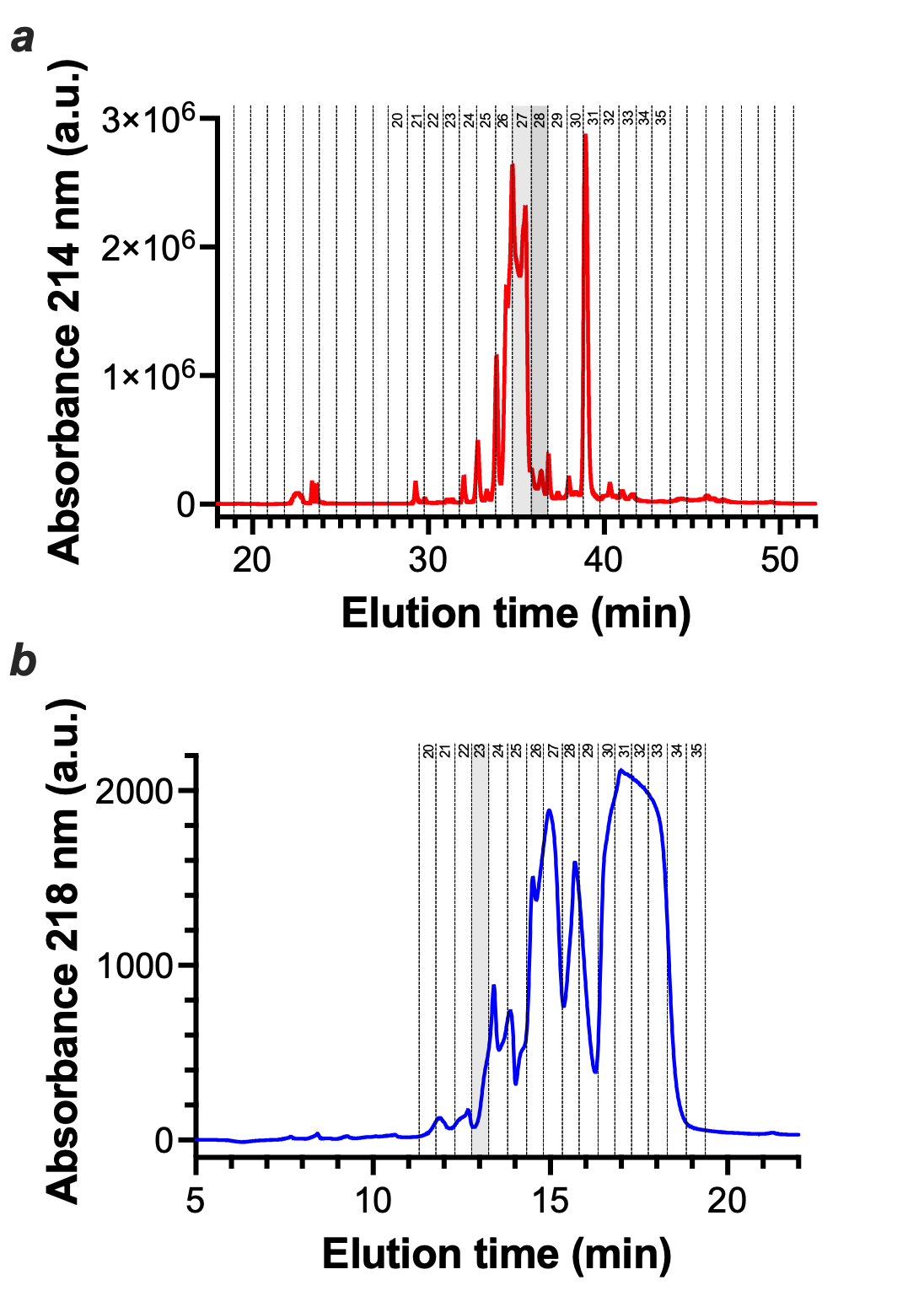
**

**Supplementary Figure 1**: HPLC fractionation of the venom of *Cyriopagopus schmidti* and purification of individual peptides. Semiprep ProteoJupiter column on Shimadzu HPLC. ***a***, RP-HPLC profile of the venom and positions of the collected fractions 27 and 28. ***b***, Cation exchange elution profile of the peptides present in fraction 27 of the RP-HPLC venom separation. SP-STAT column (Tosoh) on Agilent 1260 HPLC. Fractions were then desalted on RP Ascentis column with an Agilent 1260 HPLC. Fraction 23 highlighted in grey contains peptide F27P3.


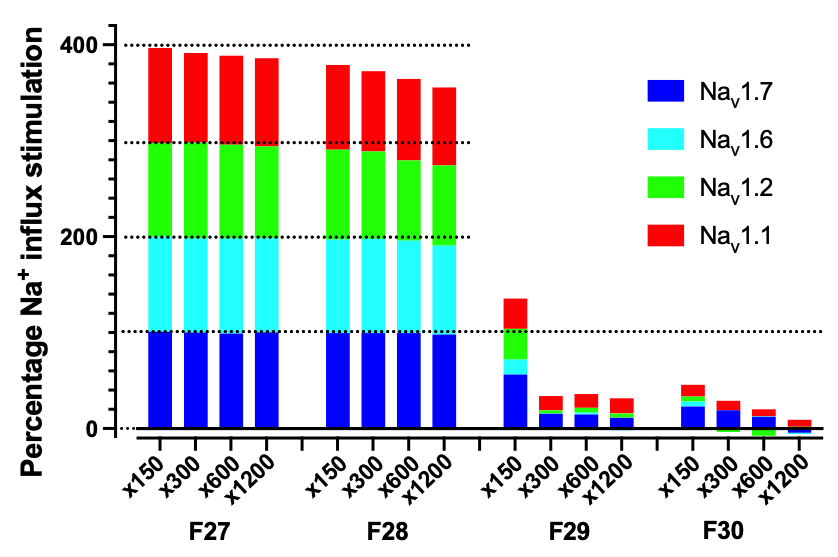


**Supplementary Figure 2**: Venom fraction dilution effect on modulation potency of four tested Na_v_ channels.


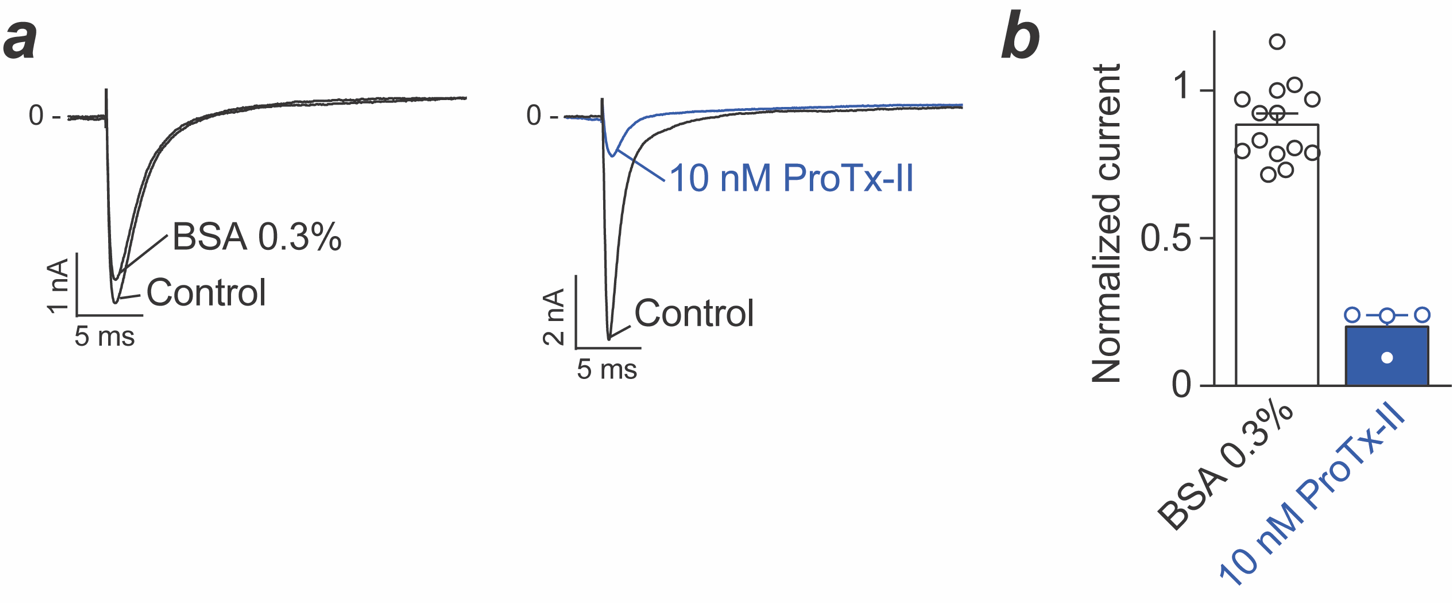


**Supplementary Figure 3**: Inhibition of Na^+^ currents by 10 nM protoxin-II from the TTX-sensitive DRG neurons studied here to investigate sHwTx-IV G_COOH_ potency. ***a***, Representative current traces before and after 10 nM protoxin-II application. ***b***, Average inhibition by 10 nM protoxin-II (n=4) compared to vehicle (n=17).


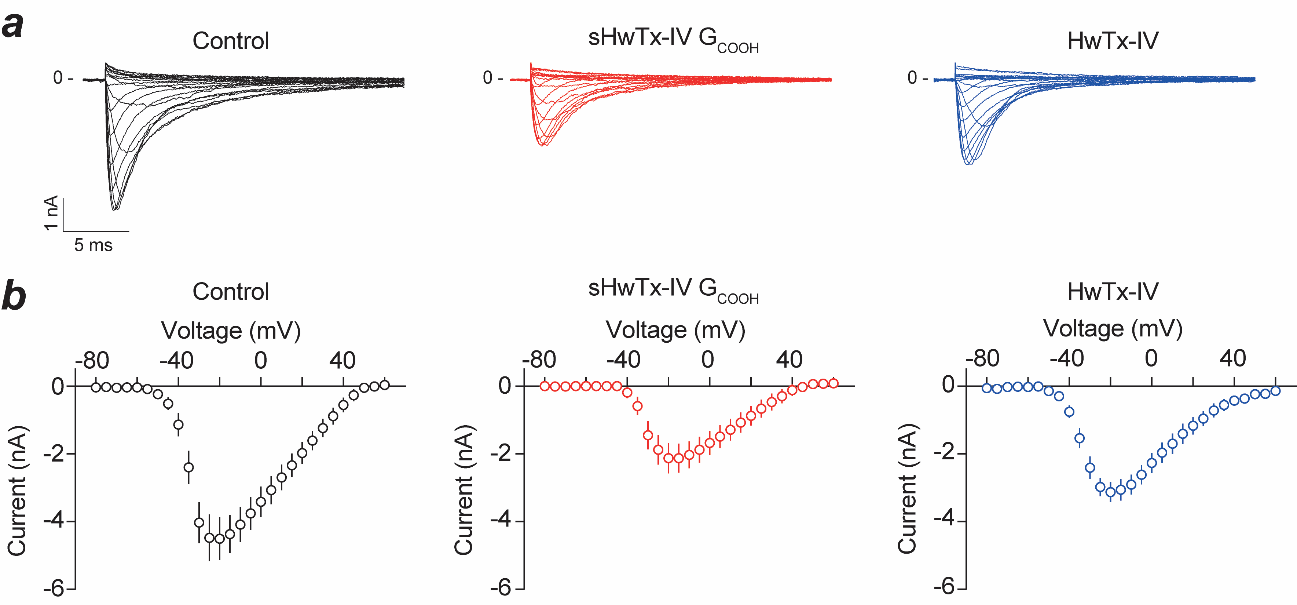


**Supplementary Figure 4**: Current-voltage curves for the inhibition of TTX-sensitive Na^+^ currents of DRG neurons by sHwTx-IV G_COOH_ or HwTx-IV. ***a***, Representative current traces, recorded during test-pulses to -80, -70, -60, -55, -50, -45, -40, -35, -30, -25, -20, -15, -10, 0, 10, 20, 30, 40, 50 and 60 mV in the absence (control) and in the presence of either 13 nM sHwTx-IV G_COOH_ or 100 nM HwTx-IV. ***b***, Average current-voltage curves under control conditions (n=12) and in the presence of either 13 nM sHwTx-IV G_COOH_ (n=5) or 100 nM HwTx-IV (n=6).

| ***Cyriopagopus schmidti* cation exchange fractions** | **MS detected (Da)** | **Peptide name** | ***Cyriopagopus schmidti* cation exchange fractions** | **MS detected (Da)** | **Peptide name** |
| --- | --- | --- | --- | --- | --- |
| F27-20/21 | 3921.7 | F27P1 | F28-2/3 | 3480.4 | F28P1 |
| F27-22 | 4143.5 | F27P2 | F28-10 | 3790.6 | F28P2 |
| F27-23 | 4161.9 | F27P3 | F28-11 | 6818.7 | F28P3 |
| F27-24 | 4161.9 + 3504.9 | F27P3 + F27P4 | F28-13 | 3779.5 | F28P4 |
| F27-25 | 3505.4 + 4085.9 | F27P4 + F27P5 | F28-16 | - | - |
| F27-26 | 4103.9 | F27P6 | F28-18 | 363.1 | - |
| F27-27 | 4103.9 | F27P6 | F28-24/25 | 3505.4 | F28P5 = F27P4 |
| F27-28/29 | 4281.0 | F27P7 | F28-32/33/34 | 3747.7 | F28P6 = F27P8 |
| F27-30/31/32/33/34 | 3747.7 | F27P8 |  |  |  |

**Supplementary Table 1**: Molecular weight of the peptides detected in the cation exchange elution fractions 20 to 34 from RP-HPLC fraction F27 and in cation exchange fractions 2 to 34 from RP-HPLC fraction 28. Eight peptides are present in fraction 27, whereas five different ones can be detected in fraction 28.
